# Adipose Tissue Dysfunction in Obesity: Role of Mineralocorticoid Receptor

**DOI:** 10.3390/nu14224735

**Published:** 2022-11-09

**Authors:** Mirko Parasiliti-Caprino, Martina Bollati, Fabio Dario Merlo, Ezio Ghigo, Mauro Maccario, Simona Bo

**Affiliations:** 1 Endocrinology, Diabetes and Metabolism, Department of Medical Sciences, University of Turin, 10126 Turin, Italy; 2 Dietetic and Clinical Nutrition Unit, Città della Salute e della Scienza Hospital, 10126 Turin, Italy; 3 Department of Medical Sciences, University of Turin, 10126 Turin, Italy

**Keywords:** mineralocorticoid receptor, obesity, insulin resistance, metabolic syndrome, adipose tissue dysfunction, oxidative stress, inflammation

## Abstract

The mineralocorticoid receptor (MR) acts as an essential regulator of blood pressure, volume status, and electrolyte balance. However, in recent decades, a growing body of evidence has suggested that MR may also have a role in mediating pro-inflammatory, pro-oxidative, and pro-fibrotic changes in several target organs, including the adipose tissue. The finding that MR is overexpressed in the adipose tissue of patients with obesity has led to the hypothesis that this receptor can contribute to adipokine dysregulation and low-grade chronic inflammation, alterations that are linked to the development of obesity-related metabolic and cardiovascular complications. Moreover, several studies in animal models have investigated the role of MR antagonists (MRAs) in preventing the metabolic alterations observed in obesity. In the present review we will focus on the potential mechanisms by which MR activation can contribute to adipose tissue dysfunction in obesity and on the possible beneficial effects of MRAs in this setting.

## 1. Introduction

Obesity, defined by the World Health Organization as an “abnormal or excessive fat accumulation that presents a risk to health” [1], is a multifactorial chronic disease associated with a reduced quality of life, a shortened lifespan, and increased healthcare costs [2,3,4]. In recent decades, its prevalence has grown to epidemic proportions [5,6], thereby becoming a major public health issue and a leading cause of morbidity and mortality worldwide [2,7]. In clinical practice, obesity is usually defined by a body mass index (BMI, i.e., weight in kilograms divided by the square of height in meters) greater than 30 kg/m^2^ [7].

The expansion of adipose tissue that is observed in obesity is the result of an imbalance between energy intake and energy expenditure and is accompanied by adipocyte dysfunction, chronic low-grade inflammation, oxidative stress, and metabolic abnormalities [8,9,10,11,12]. Not only the amount of excess fat, but more specifically, the distribution of adipose tissue within the body, has an important role in determining the risk of obesity-related health complications. Visceral adiposity, rather than subcutaneous adiposity, is more strongly associated with the development of insulin resistance, type 2 diabetes mellitus (T2DM), and metabolic syndrome, as well as nonalcoholic fatty liver disease/steatohepatitis, arterial hypertension, and cardiovascular complications [7,13,14,15,16].

The mineralocorticoid receptor (MR) is an intracellular transcription factor that represents the final effector of the renin–angiotensin–aldosterone system and acts as a key regulator of blood pressure, electrolyte balance, and volume status in the kidney [17,18]. In recent decades, the identification of MR in other organs, such as the vasculature, the heart, the adipose tissue, the brain, and the immune system, has shifted the attention to the so-called “non classical” effects of MR. Indeed, the activation of MR has been shown to promote inflammation, oxidative stress, and fibrosis in several target tissues, thereby leading to an increased risk of cardiovascular, metabolic, and renal complications [18,19,20,21].

The discovery of the MR receptor in adipose tissue, together with the finding that MR is overexpressed in adipose tissue of patients with obesity [22,23], has led to the hypothesis that MR can contribute to adipose tissue dysfunction and obesity-related complications by promoting inflammation, oxidative stress, and adipokine dysregulation. This may have potential therapeutic implications, suggesting a possible role of mineralocorticoid receptor antagonists (MRAs), such as spironolactone, eplerenone, canrenone, and potassium canrenoate in obese patients. Often adopted for the treatment of arterial hypertension or heart failure, these drugs could be effective in ameliorating metabolic parameters in patients with obesity [24,25,26].

In the present review, we will focus on the potential role of MR in the pathophysiology of adipose tissue dysfunction and in the development of obesity-related cardiovascular and metabolic complications.

## 2. Materials and Methods

We conducted a literature search in PubMed, the Cochrane Library, EM-BASE, and CINAHL databases to search for studies on the role of MR in obesity, combining MR and obesity/adipose tissue/metabolic syndrome/visceral adiposity. No restrictions were imposed, apart from the English language. The search strategy also included the accurate evaluation of the references of the studies on the field. Systematic reviews and meta-analyses were considered with priority, followed by randomized controlled trials and thereafter, by observational studies and case series. Considering the topic of this review, animal and in vitro studies were included.

## 3. Adipose Tissue

Traditionally, adipose tissue has been considered a static depot of energy. When calorie intake exceeds the requirements of the body, the excess energy is stored in lipidic droplets in the form of triglycerides thanks to the activity of lipogenic enzymes. Conversely, in conditions of increased energy expenditure, free fatty acids are released by the action of lipolytic enzymes to provide fuel to other organs [27]. In recent decades, however, it has become clear that adipose tissue is much more than an inert energy storage and that it rather represents a dynamic and metabolically active organ composed of a vast heterogeneity of cell types with important immune and endocrine functions [28,29].

From a histological point of view, adipose tissue is a connective tissue made of different cell types (adipocytes, preadipocytes, mesenchymal stem cells, endothelial cells, pericytes, nerve fibers, and immune cells) of which adipocytes represent by far the predominant cell type. Based on adipocyte characteristics, adipose tissue can be further classified in white adipose tissue (WAT) and brown adipose tissue (BAT) with distinct morphology, function, and embryologic origin [30,31].

WAT consists of unilocular, rounded cells derived from myogenic factor 5 (Myf5)-negative progenitors that contain a single large lipid droplet and a flattened peripheral nucleus [32]. Based on its location within the body, WAT can be further divided into visceral adipose tissue (VAT), which in humans is mainly located in the mesentery and omentum, and subcutaneous adipose tissue (SAT), located beneath the skin [30,31]. The expansion of VAT has been strongly linked to the development of obesity-related metabolic abnormalities such as insulin resistance, diabetes mellitus, metabolic syndrome, and dyslipidemia [33,34,35]. As regards SAT, data are not conclusive, suggesting significant differences in the functional characteristics of different fat depots: whereas abdominal/truncal SAT has been associated with insulin resistance, some authors have hypothesized that gluteo-femoral SAT may have a protective role [34,35,36].

Besides being able to store energy inside their lipid droplets, white adipocytes can secrete more than 600 different types of molecules, the so-called adipokines, which are involved in the regulation of a variety of metabolic and immune processes [37]. Adipokines form a heterogeneous group of molecules that comprises hormones, cytokines, growth factors, vasodilators, and several other substances; the detailed description of the specific functions of different adipokines, however, goes beyond the purpose of this review and is detailed elsewhere [37,38]. Leptin was the first adipocyte-secreted hormone to be discovered [39]. This protein is released by white adipocytes proportionally to WAT mass and reflects the status of energy depots. It regulates the sense of satiety, reducing appetite and food intake, and ameliorates insulin sensitivity. Mice with loss of function mutations in the gene-encoding leptin or leptin receptor (ob/ob and db/db mice, respectively) are used as a model of obesity and T2DM. Adiponectin, another adipokine secreted by WAT adipocytes, exerts an insulin-sensitizing and anti-inflammatory function, by inhibiting the production of pro-inflammatory cytokines, as well as monocytes’ adhesion to endothelial cells. Other secretory proteins, such as monocyte chemoattractant protein-1 (MCP-1), regulate the recruitment of immune cells, whereas interleukin-1β (IL-1β), interleukin-6 (IL-6), plasminogen activator inhibitor-1 (PAI-1), and tumor necrosis factor-α (TNF-α) contribute to the inflammatory process [38].

BAT is composed of multilocular cells with a rounded central nucleus, multiple cytoplasmic droplets, and cristae-enriched mitochondria that express, among others, the uncoupling protein 1 (UCP-1) on their inner membrane. This protein promotes thermogenesis by uncoupling oxidative phosphorylation and adenosine triphosphate (ATP) synthesis, thereby dissipating energy in the form of heat. BAT adipocytes derive from the same precursors of skeletal muscle cells (Myf5-positive embryonic precursors) and are involved in non-shivering thermogenesis [32]. Recent studies suggest that metabolically active BAT can be found in human adults (mainly, around the neck and large blood vessels of the thorax) and its extension is inversely correlated with BMI, indicating that increased amounts of BAT may protect from obesity [40,41,42,43,44]. Indeed, BAT activation has been shown to improve metabolic parameters, with increased glucose uptake and insulin sensitivity, as well as improved clearance of plasma triglycerides and cholesterol, possibly protecting from atherosclerotic plaque formation [45]

Finally, a third type of adipocytes, the so-called beige or “brite” adipocytes, represent those adipocytes interspersed between WAT that can acquire a “brown-adipocyte phenotype” as a result of cold exposure or adrenergic stimulation, even if they derive from Myf5-negative precursors [32]. It is not clear whether beige adipocytes derive from transdifferentiation of white adipocytes or from de novo differentiation of precursors [32].

Besides adipocytes, adipose tissue is composed of resident immune cells of which adipose tissue macrophages (ATMs) are the most numerous cell type. They constitute highly plastic cells, whose immunophenotype changes in response to different stimuli in their surrounding microenvironment. In lean subjects, they represent around 5% of adipose tissue cells and play a key role in maintaining tissue turnover by engulfing dead adipocytes. Moreover, they can act as lipid buffers: in conditions of increased lipolysis, they take up free fatty acids (FFA), thus reducing their release in the bloodstream [46].

## 4. Adipose Tissue Dysfunction in Obesity

As we have already mentioned, obesity is characterized not only by an abnormal enlargement of adipose tissue due to calorie excess, but also by significant morphologic and functional changes in fat depots that are responsible for obesity-related metabolic complications [9]. More specifically, VAT expansion, rather than SAT expansion, is more strongly associated with the development of insulin resistance, metabolic syndrome, and cardiovascular complications [7,13,15,16,33].

Adipose tissue enlargement may occur either by recruitment and proliferation of pre-adipocytes (hyperplasia) or by enlargement of existing adipocytes (hypertrophy). In obesity, the main mechanism underlying adipose tissue expansion is hypertrophy [47]. Hypertrophic adipocytes display an enhanced expression of pro-inflammatory adipokines, such as IL-6, PAI-1, TNF-α, and MCP-1 and a reduced expression of anti-inflammatory and insulin-sensitizing adipokines (adiponectin, interleukin 10 [IL-10]). This leads to insulin resistance and recruitment of macrophages, contributing to the development of low-grade inflammation [8,9,46,47]. 

Another pro-inflammatory stimulus is represented by oxidative stress, which is increased in adipocytes as a result of mitochondrial dysfunction and increased activity of nicotinamide adenine dinucleotide phosphate (NADPH) oxidase (NOX) [47]. In the presence of high levels of ROS, nuclear factor kappa-light-chain-enhancer of activated B cells (NF-kB) is released from its repressor NF-kB inhibitor ⍺ (IkB⍺) and can move to the nucleus, where it binds to specific sequences of DNA called response elements, thereby inducing the transcription of pro-inflammatory and pro-fibrotic genes [19]. 

In addition, hypertrophy also impairs blood supplies, leading to local hypoxia and increased expression of hypoxia inducible factor 1-α (HIF1-α), another pro-inflammatory and pro-fibrotic factor [46]. Eventually, low-grade chronic inflammation and adipokine dysregulation lead to the development of insulin resistance and metabolic syndrome [8,9,47]. 

Moreover, in hypertrophic adipocytes, basal lipolysis is increased, leading to the leakage of FFA that in turn can contribute to fat accumulation and lipotoxicity in other tissues [8,9,47]. Ectopic fat accumulation in the heart, pancreas, liver, kidney, and skeletal muscle has been indeed associated with chronic inflammation and with the development of cardiometabolic and renal complications [9,48]. 

**Figure 1:**
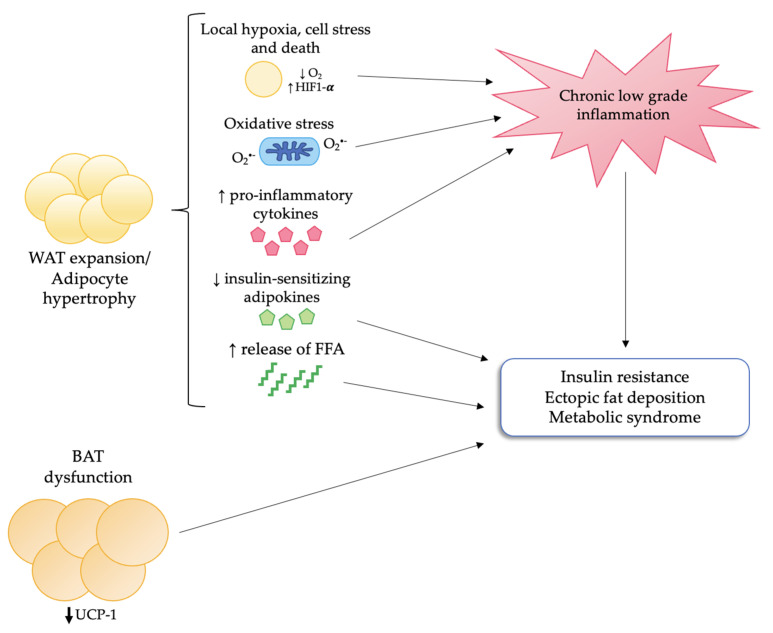
**Adipose tissue dysfunction in obesity.** In obesity, WAT responds to calorie overload mainly through adipocyte hypertrophy. This leads to local hypoxia, cell stress and death, oxidative stress, and increased secretion of pro-inflammatory cytokines with subsequent infiltration of macrophages and development of chronic low-grade inflammation. Moreover, hypertrophic adipocytes display an impaired secretion of insulin-sensitizing molecules, such as adiponectin, and an increased release of FFA. All these changes, together with BAT dysfunction and impaired browning of WAT, eventually lead to insulin resistance, ectopic fat deposition, and metabolic syndrome. Abbreviations: BAT, brown adipose tissue; FFA: free fatty acids; HIF-1α: hypoxia inducible factor-1α; UCP-1: uncoupling protein-1; WAT: white adipose tissue.

Finally, impaired BAT function may contribute to the development of metabolic alterations such as insulin resistance and dyslipidemia. Figure 1 summarizes the main mechanisms underlying adipose tissue dysfunction in obesity.

## 5. MR and Adipose Tissue Dysfunction

MR is a member of the nuclear receptor family that binds with equal affinity mineralocorticoids (i.e., aldosterone) and glucocorticoids (i.e., cortisol). Upon the binding of its ligand, MR homodimerizes and moves to the nucleus, where it binds to hormone response elements located in the promoter of target genes, thereby acting as a transcription factor [18]. 

MR is expressed in several tissues, such as the kidney and other epithelial tissues (colon, salivary glands) where it acts as the final effector of the renin–angiotensin–aldosterone system, determining the reabsorption of sodium and water and the excretion of potassium. MR is therefore a key regulator of volume status, blood pressure, and electrolyte balance. Notably, in the kidney, the selective response of MR to aldosterone depends on the activity of 11-β-hydroxysteroid-dehydrogenase type 2 (11β-HSD2), an enzyme which selectively inactivates glucocorticoids [18]. 

In the last decades, MR has been discovered in several non-epithelial tissues such as heart, blood vessels, immune cells, neurons, and adipose tissue. Since glucocorticoids circulate at 100 to 1000-fold higher concentration than those of aldosterone; in these tissues, MR is mainly activated by cortisol due to the absence of 11β-HSD2 [26]. However, other mechanisms that prevent MR activation by glucocorticoids might occur in non-epithelial tissues. For example, levels of bioactive cortisol may be reduced by its binding to plasma proteins (albumin and corticosteroid binding protein), whereas circulating aldosterone is mainly found in a free form. Furthermore, a change in the redox status of the cell may regulate cortisol-mediated MR activation [25]. Finally, in pathological conditions, local expression of either aldosterone synthase or 11β-HSD2 may be induced [49,50].

Besides its well-known effect on electrolyte and water homeostasis, growing attention has been directed to the so-called “non-classical” effects of MR activation. Indeed, it has been demonstrated that MR is involved in pro-inflammatory, pro-oxidative, and pro-fibrotic changes in various target tissues, and several studies have proposed its causative role in different cardio-metabolic diseases [18,19]. 

MR expression has been demonstrated both in BAT and WAT and seems to have a key role in pre-adipocyte differentiation, driving the acquisition of a mature phenotype [51]. More specifically, in a study by Caprio et al., exposure of cultured 3T3-L1 cells to aldosterone led to an increase in intracellular lipid content and induced the expression of markers of adipocyte conversion, such as adiponectin, leptin, and resistin, driving the acquisition of a white adipocyte phenotype. Moreover, aldosterone stimulated the expression of peroxisome proliferator-activated receptor γ (PPAR-γ) which acts as a crucial transcriptional regulator of adipogenesis. MR inactivation either with spironolactone or specific small interfering ribonucleic acids (siRNA) reversed these effects [51]. Similar results were obtained in BAT by Hoppman et al., who demonstrated that MR knockout in pre-adipocytes prevented the formation of lipid droplets, whereas glucocorticoid receptor (GR) knockout only mildly affected adipogenesis [52]. 

These findings have led to the hypothesis that MR may represent an important regulator of metabolic homeostasis both in physiologic and pathologic conditions. 

Focusing our attention on the potential role of MR in obesity, several studies in animal models have highlighted that MR is overexpressed in adipose tissue of subjects with obesity. In 2009, Hirata et al. demonstrated that mRNA levels of MR were significantly higher in WAT of db/db and ob/ob mice compared with lean controls [53]. A more recent study by the same group confirmed that MR was overexpressed in db/db mice, whereas the expression of GR was not significantly increased [23].

In a subsequent study by Urbanet et al., the authors showed that MR mRNA was significantly higher in VAT when compared with SAT. Moreover, MR mRNA levels were higher not only in SAT and VAT of db/db mice but also in adipose tissue of patients with obesity as compared with lean controls. Finally, the same authors found that mice overexpressing MR were more prone than controls to gain body weight due to an increase in WAT mass with concurrent adipocyte hypertrophy [22]. 

High levels of ROS, increased activity and expression of 11-β-hydroxysteroid-dehydrogenase type 1 (11β-HSD1), and high plasma levels of aldosterone have been suggested as causes of MR overactivity in patients with obesity, but the exact mechanisms underlying this phenomenon are yet to be elucidated [53].

The finding that adipocyte MR is overexpressed in subjects with obesity as well as in VAT when compared with SAT has led to the hypothesis that this receptor may also be involved in obesity-related adipocyte dysfunction, low-grade inflammation, and obesity-related metabolic complications.

### 5.1. MR and Inflammation

As we have already mentioned, obesity is characterized by a state of chronic, low-grade inflammation that is responsible for the development of obesity-related complications, since increased levels of pro-inflammatory cytokines, such as C reactive protein, IL-6, and PAI-1, are associated with impaired insulin sensitivity and increased risk of T2DM and metabolic syndrome in humans [54,55,56]. An increase in adipocyte size can lead to dysregulation of adipokine secretion. In particular, the production of pro-inflammatory cytokines is considerably enhanced in hypertrophic adipocytes [57]. Treatment of obese mice with MRAs is associated with a reduction of adipocyte volume, which seems to be mediated, at least in part, by the upregulation of PPAR-γ. The reduction in adipocyte size is accompanied by a change in the secretory pattern of the cell, with an increase in adiponectin levels and a reduction in pro-inflammatory cytokines such as TNF-alfa, MCP-1, and IL-6 [53]. 

The crosstalk between hypertrophic dysfunctional adipocytes and ATMs has a crucial role in determining the pro-inflammatory state observed in obesity. The number of ATMs increases dramatically in obesity both in mouse models and in humans, reaching up to 50% of all adipose tissue cells [46]. The increase in ATMs number mostly results from an increased production of MCP-1 and other chemotactic factors released by dysfunctional hypertrophic adipocytes, which in turn leads to the recruitment of monocytes from the bloodstream. This is accompanied by a shift in macrophage polarization from the so-called alternatively activated M2 phenotype to classically activated M1 phenotype. In lean subjects, the M2 macrophages, which are characterized by the expression of anti-inflammatory molecules, such as IL-10, are the prevailing phenotype. Vice versa, in patients affected by obesity, macrophages undergo a polarization shift to a pro-inflammatory M1 phenotype and start expressing pro-inflammatory cytokines such as IL-6, TNF-α, MCP-1, etc. [9]

Another major stimulus for the recruitment of inflammatory cells in adipose tissue is adipocyte stress and death, which is in turn linked to lipid overload and adipocyte hypertrophy. Macrophages accumulate around the dying adipocyte, forming so-called crown-like structures (CLS), whose number is increased in patients with obesity [58,59].

Finally, adipocyte hypertrophy can impair blood supplies causing local hypoxia, which in turn triggers inflammation by inducing the expression of HIF1-α, a transcriptional factor that increases the expression of pro-inflammatory cytokines (PAI-1, IL-6, vascular endothelial growth factor [VEGF], etc.) and reduces the expression of adiponectin, which has an anti-inflammatory effect. Local hypoxia, together with increased release of FFA from hypertrophic adipocytes, can also induce mitochondrial dysfunction in ATMs, leading to an increased production of reactive oxygen species (ROS) that further triggers the activation of pro-inflammatory genes [60,61].

The pro-inflammatory effects of MR-activation have been demonstrated in several target organs, including the heart, the vasculature, and the kidney [18,19]. Several studies on cultured adipocytes and animal models have confirmed that MR can also promote inflammation in adipose tissue. 

In a study by Hoppman et al., selective MR stimulation with aldosterone promoted a pro-inflammatory and diabetogenic adipokine expression profile in cultured adipocytes, leading to an increased expression of IL-6, PAI-1, MCP-1, and chemerin [52].

In another study by Guo et al., undifferentiated 3T3-L1 preadipocytes exposed to aldosterone showed a 6-fold increase in mRNA levels of TNF-α, a 2-fold increase in IL-6 and MCP-1, and a decrease in molecules involved in the downregulation of inflammatory response, such as PPAR-γ and adiponectin. These effects were prevented by MR antagonism with canrenoate [62]. 

Moreover, in a study by Labuzek et al. in human monocyte-derived macrophages, treatment with eplerenone affected the expression of arginase I and mannose receptor, marker of M2 phenotype, changes that were similar to those produced by IL-4, a cytokine that is known to induce alternative activation in macrophages [63]. 

Finally, Wada et al. showed that treatment with eplerenone decreases macrophage infiltration and CLS formation in mice fed with a high fat diet (HFD). Furthermore, eplerenone attenuates M1-polarization of macrophages and inhibits the activation of NLR family pyrin domain containing 3 (NLPR3)-inflammasome, a key mediator of the inflammatory response [64]. 

**Figure 2:**
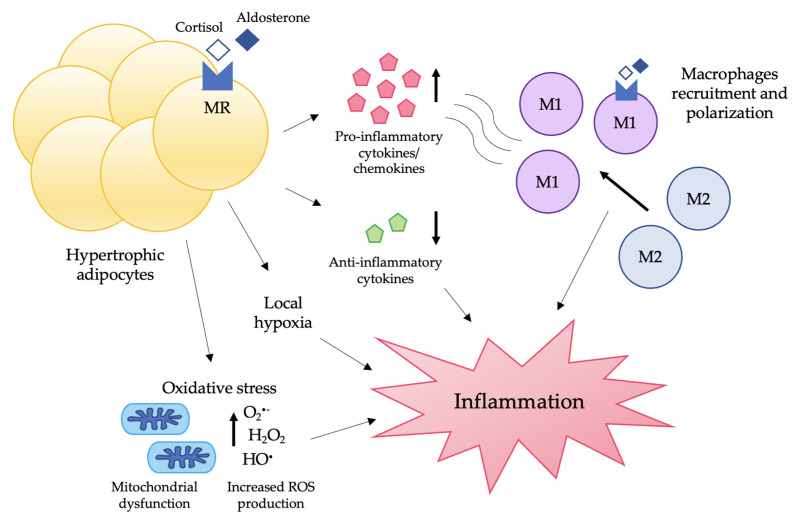
**Contribution of MR to adipose tissue inflammation.** The activation of MR, either by aldosterone or by cortisol, leads to adipocyte hypertrophy, which in turn causes cell stress and death and local hypoxia. Moreover, hypertrophic adipocytes display an impaired secretion of adipokines, with an increased release of pro-inflammatory molecules. These changes promote the infiltration of macrophages and their polarization shift to a M1 phenotype. Finally, MR activation is linked to mitochondrial dysfunction and enhanced ROS production, which contribute to oxidative stress, another important pro-inflammatory stimulus. Abbreviations: MR, mineralocorticoid receptor; ROS: reactive oxygen species.

In conclusion, MR activation in adipocytes is linked to an increased secretion of pro-inflammatory cytokines and chemokines, with the subsequent recruitment of immune cells from the bloodstream. Moreover, MR activation can directly stimulate a shift from a M2 to a M1 phenotype in macrophages (Figure 2). Finally, MR activation can promote inflammation by increasing ROS production, as will be discussed in the following section.

### 5.2. MR and Oxidative Stress

ROS, such as superoxide anion, hydrogen peroxide, and hydroxyl radical, are highly reactive compounds derived from oxygen. The main endogenous source of ROS is the mitochondrial oxidative phosphorylation, but they may also be generated as by-products of other chemical reactions, such as the one catalyzed by NOX in immune and endothelial cells, as well as by the interaction with a variety of xenobiotics [65]. 

In physiologic conditions, ROS are involved in many cellular signaling pathways that regulate proliferation, differentiation, apoptosis, and immune response. However, when their production overwhelms the cellular antioxidant defense mechanisms, oxidative stress occurs, resulting in ROS-mediated damage of nucleic acids, proteins, and lipids [65]. Oxidative stress has been implicated not only in aging, but also in the pathogenesis of a wide variety of pathological conditions (tumorigenesis, neurodegenerative diseases, atherosclerosis, diabetes, and metabolic syndrome) [66,67,68,69,70].

The role of oxidative stress in obesity is currently well established. Subjects with obesity display mitochondrial dysfunction with enhanced ROS production as compared to lean subjects. In 2004, Furukawa and colleagues showed that in individuals affected by obesity, plasma thiobarbituric acid reactive substance (TBARS), a marker of oxidative injury, significantly correlated with BMI and waist circumference. The authors also demonstrated that plasma markers of oxidative stress were higher in obese mice as compared to lean mice, independent of hyperglycemia. In WAT of obese mice, lipid peroxidation and H_2_O_2_ production, both signs of oxidative stress, were elevated and increased oxidative stress in this tissue was due both to NOX induction and to impaired antioxidant defense mechanisms [71]. 

In another study by Hirata et al., authors showed increased expression of NOX subunits and decreased expression of ROS-scavenging enzymes, such as catalase and Cu, Zn-superoxide dismutase, in obese mice as compared to lean control mice [53]. 

Furthermore, Chattopadhyay et al. showed that mitochondria from patients with obesity produced significantly higher levels of ROS in vitro as compared to controls, suggesting that mitochondria dysfunction has an important role in obesity-related oxidative stress [72]. 

In the last decades, several studies have suggested that MR activation by aldosterone can promote oxidative stress and mitochondrial dysfunction in several target tissues. Upon binding to MR, aldosterone can induce the activation of NOX in several cell types, such as endothelial cells, cardiomyocytes, mesangial cells, podocytes, and macrophages, thereby leading to increased production of anion superoxide [19]. Another way by which MR activation can lead to oxidative stress is through the uncoupling of nitric oxide synthase, which leads to the generation of superoxide instead of nitric oxide in endothelial cells [73]. Moreover, MR activation can reduce the expression glucose-6-posphate dehydrogenase in vascular cells, thus impairing the generation of NADPH, which is in turn required for the production of reduced glutathione, one of the main defenses of the cell against oxidative stress [74]. Finally, aldosterone binding to the MR was shown to promote mitochondrial dysfunction, further contributing to the generation of ROS and leading to cell damage, an effect that has been extensively studied in podocytes [75,76].

Focusing our attention on adipose tissue, several studies have demonstrated a role of MR in mediating oxidative stress in adipocytes.

In a study by Hirata et al., MR blockage either with eplerenone or small interfering RNA (siRNA) blunted aldosterone-mediated increase in ROS production by reducing the expression of subunits p22 and p47 of NOX and by raising the levels of antioxidant enzymes such as catalase and Cu, Zn-superoxide dismutase, both in obese mice and in 3T3-L1 adipocytes [53]. A more recent study by the same group confirmed these results, showing increased levels of TBARS, a marker of oxidative stress, as well as a significant increase in mRNA levels of NOX subunit p22 and a decrease in catalase mRNA level in adipocytes exposed to glucocorticoids. These changes were reversed by eplerenone, thereby suggesting a MR-mediated mechanism [23].

In another study by Lefranc et al., the authors investigated the MR-mediated regulation of mitochondrial respiration in adipose tissue both in vivo and in vitro, demonstrating that MR activation increases the production of ROS and causes mitochondrial dysfunction, whereas treatment with MRAs improves obesity-related oxidative stress and prevents cell senescence. In this study, aldosterone increased H2O2 levels and oxygen consumption rate, a marker of mitochondrial respiration, whereas MR antagonism prevented these effects both in db/db mice and in 3T3-L1 adipocytes. Moreover, MR antagonism prevented cell senescence through the downregulation of p53-p21 pathway and the upregulation of sirtuins, deacetylase, which are involved in cell survival [77]. 

In line with these findings, in a more recent study by the same group, the authors demonstrated that MR-overexpression in mice leads to mitochondrial dysfunction and premature senescence in adipocytes. More specifically, MR overexpression is associated with an increase in levels of superoxide and hydrogen peroxide in epididymal VAT. Moreover, mitochondria of mice overexpressing MR display a reduced ability to respond to increased energy requirements as compared with controls. Finally, MR overactivation upregulates mitophagy (i.e., mitochondrial-targeted autophagy that enables selective degradation of damaged mitochondria) and inhibits the synthesis of new molecules of mitochondrial DNA. All these MR-mediated changes in mitochondrial function eventually cause adipocyte dysfunction and senescence [50].

### 5.3. MR Activation in BAT

The finding that transgenic mice with primary deficiency of BAT or lack of UCP-1 were prone to develop obesity, together with the evidence that BAT activation was associated with improved insulin-sensitivity both in humans and in animal models, has led to the hypothesis that stimulating BAT activity might be a promising strategy in preventing fat mass accumulation and insulin-resistance in obesity [32,78,79]. 

The expression of MR in BAT was first demonstrated by Zennaro et al. in 1998. The authors developed a transgenic mouse model expressing the SV40 large T4 antigen (an oncoprotein that can induce malignant transformation of a variety of cell types) under the control of the two promoters of the human MR gene (P1 and P2). The finding that these mice developed malignant hibernoma (brown fat tumor) demonstrated that the human MR promoter P1 was transcriptionally active in BAT adipocytes and therefore suggested a potential role for MR in adipocyte differentiation [80].

In a subsequent study conducted by Penfornis et al. on a brown adipocyte cell line derived from a hibernoma of transgenic mice carrying the proximal promoter of the human MR connected to the SV40 large T antigen (T37i cells), aldosterone treatment of undifferentiated cells resulted in accumulation of intracytoplasmic lipid droplets and mitochondria, as well as in a significant increase in intracellular triglyceride content. These effects were abolished by the presence of MR antagonists, thereby suggesting the involvement of MR in the process. In the same study, the expression of early adipogenic gene markers such as lipoprotein lipase, PPAR-γ, and adipocyte-specific fatty acid binding protein 2 was increased by aldosterone. In light of these results, the authors concluded that MR-activation was involved in the early induction of brown adipocyte differentiation [81].

In a later study by Viengchareun et al., the authors demonstrated that MR activation inhibits the expression of UCP1 and UCP3 which, as we have already mentioned, are the key enzymes in non-shivering thermogenesis [82]. Similar findings were outlined in a study by Kraus et al., in which aldosterone dose-dependently inhibited the expression of UCP-1 by 30% in BAT [83]. 

MR overactivation has also been shown to impair β3-adrenergic receptor response in mouse-derived brown adipocytes with a consequent decrease in oxygen consumption, lipolysis, and UCP-1 expression, effects that appears to be mediated by the interaction with Twik-related acid-sensitive K+ channel 1 (TASK-1) [84]. 

Accordingly, in a more recent study by Kuhn et al., cold-induced expression of UCP-1 in BAT was blunted in MR-overexpressing mice [85].

Moreover, in a study by Armani et al. in mice fed with a HFD, treatment with MR antagonists induced browning of WAT, increasing UCP-1 protein expression, with a concomitant increase of glucose uptake in adipose tissue. This process was associated with a reduced rate of autophagy [86]. 

Furthermore, in a study by Marzolla et al. in mice fed with a HFD, the MR antagonist finerenone led to an increase in BAT density in interscapular fat and to a higher expression of thermogenesis-related markers in BAT (i.e., UCP-1, peroxisome proliferator-activated receptor-gamma coactivator 1-α (PGC1-α), and β-3 adrenoreceptor). These changes were associated with an improvement in metabolic parameters and glucose tolerance. Moreover, in T37i brown pre-adipocytes, finerenone was able to stimulate thermogenesis via adenosine monophosphate-activated protein kinase (AMPK), inducing the activation of triglyceride lipase and increasing the expression of UCP-1 [87]. 

These results were confirmed in a more recent study by the same group, in which finerenone but not spironolactone, increased the recruitment of brown adipocytes as well as the expression of UCP-1, PGC1-α, and β-3 adrenoreceptor in interscapular BAT of very HFD-fed mice, with a concurrent improvement in insulin resistance as indicated by a significant reduction of HOMA-IR index. The authors hypothesize that the different pharmacokinetics of the two drugs may influence their distribution in BAT, thereby explaining a higher responsiveness of this tissue to finerenone [88]. 

Finally, in a randomized double-blind placebo-controlled study by Thuzar et al., MR antagonism enhanced BAT function in response to cold exposure and to a mixed meal in humans. More specifically, MR antagonism with spironolactone blunted the decrease in skin temperature overlying supraclavicular BAT during cooling and increased postprandial heat production, with a concomitant reduction in lipid synthesis. According to the authors, this change in energy usage from storage to dissipation as heat suggests that MR antagonism may be a promising therapeutic strategy in obesity [89]. 

All together, these findings suggest that MR activation inhibits heat production in BAT in favor of energy storage. By promoting energy dissipation as heat in BAT and possibly by favoring browning of WAT, MR antagonism may therefore prevent excessive lipid accumulation and improve metabolic parameters. 

### 5.4. MR, Insulin Resistance and Metabolic Syndrome

Visceral obesity is associated with insulin resistance, T2DM, and metabolic syndrome. As we have already mentioned, the development of insulin resistance in patients with obesity is related to adipokine dysregulation secondary to adipocyte hypertrophy and dysfunction, which leads to a decrease in insulin-sensitizing molecules such as adiponectin, and to an increase in pro-inflammatory cytokines [37,38,90].

Several studies in animal models have suggested an important role of MR in the development of obesity-related insulin resistance and an insulin-sensitizing effect of MRAs. As shown by Urbanet et al., mice with selective overexpression of adipocyte-MR display multiple metabolic abnormalities including visceral obesity, insulin resistance, and dyslipidemia [22]. 

In a study by Guo et al. in db/db mice, treatment with eplerenone was associated with an improvement of homeostasis model assessment for insulin resistance (HOMA-IR) index, a widely used indicator of insulin resistance, and with a reduction in plasma triglycerides levels, with a concomitant increase in adiponectin [62]. 

Similarly, Hirata et al. showed a significant decrease in plasma glucose and triglyceride levels and an improvement in HOMA-IR in ob/ob mice and db/db mice treated with eplerenone, changes that were accompanied by an increase in plasma levels of adiponectin. Moreover, eplerenone blunted the elevation of plasma glucose during glucose tolerance test and decreased plasma glucose levels after insulin tolerance test, suggesting an improvement in insulin sensitivity [53]. 

In addition, in a study by Armani et al., the MR antagonism with either spironolactone or drospirenone prevented an HFD-induced blood glucose increase after an intraperitoneal glucose tolerance test in HFD-fed mice. This improvement in glucose tolerance was accompanied by a concomitant browning of VAT, suggesting that this process may have a beneficial role in preserving and/or restoring insulin sensitivity [86]. 

Accordingly, in a more recent study by Marzolla et al., finerenone improved glucose tolerance in HFD-fed mice, as shown by a significant reduction in plasma glucose after intraperitoneal glucose tolerance test. These changes were associated with enhanced expression of thermogenesis-related markers in interscapular BAT [87]. 

Moreover, in a study by Wada et al., eplerenone attenuated body weight gain, with a reduction both in SAT and VAT, and improved glucose metabolism, as well as blood pressure and cholesterol levels in mice fed with an HFD. Eplerenone treatment also decreased lipid accumulation and triglyceride content in hepatic lobules [64].

Furthermore, a study by Zhang et al. suggested a role of macrophage MR in the development of insulin resistance in obese mice, since the myeloid-specific MR blockade improved insulin sensitivity and hepatic lipid metabolism [91].

Finally, in a recent study by Bavuu et al., aldosterone impaired insulin signaling in cultured adipocytes, as demonstrated by a reduction in insulin-induced Akt-phosphorylation, and reduced the expression of insulin sensitivity-associated genes, such as adiponectin and PPAR-γ. On the contrary, treatment with esaxerenone, a novel MRA, was associated with an amelioration of insulin sensitivity and an increased expression of adiponectin and PPAR-γ both in cultured adipocytes and HFD-fed mice [92]. 

In contrast with the aforementioned studies, Homma et al. demonstrated that treatment with spironolactone, but not with eplerenone, was associated with an increase in plasma blood glucose in basal conditions and after oral glucose tolerance test, without changes in the levels of insulin and adiponectin in a rat model of metabolic syndrome [93]. The authors suggested that the different effect of these two MRAs on glucose metabolism may be related to the lower selectivity of spironolactone for MR, since this drug also binds glucocorticoid, androgen, and progesterone receptors with a higher affinity than eplerenone [93].

Clinical data on the potential benefit of MRA treatment on insulin resistance are not conclusive. Serum aldosterone levels have been associated with the development of the metabolic syndrome, as suggested by the Framingham Offspring Study [94]. Accordingly, primary aldosteronism, a condition of aldosterone excess that determines MR-overactivation, has been linked with an increased risk of T2DM and metabolic syndrome [95]. In addition, in patients with primary aldosteronism, treatment with adrenalectomy has been shown to improve insulin sensitivity, whereas data regarding the effect of MRA treatment on glucose metabolism in this population are conflicting [96,97,98,99].

Shifting our attention on patients with metabolic syndrome, Derosa et al. have shown that MR antagonism with canrenone significantly decreases fasting plasma insulin and improved HOMA-IR index, a widely used marker of insulin resistance [100]. 

Conversely, studies in healthy subjects and in normotensive, non-diabetic patients with obesity, showed no significant effect of spironolactone on insulin sensitivity [101]. These conflicting results in humans may be related to the heterogeneity of the populations studied and to the different treatment protocols. Therefore, further studies on selected populations are needed to clarify the real potential of MRA in the improvement of metabolic parameters in subjects with obesity.

## 6. Conclusions

Obesity is a complex multifactorial disease characterized not only by the abnormal expansion of fat depots, but also by a pro-inflammatory and pro-oxidative environment, as well as by adipocyte dysfunction, which are in turn responsible for obesity-related metabolic and cardiovascular complications.

In recent decades, a growing body of evidence has highlighted the detrimental role of MR-overactivation in several target organs, including adipose tissue, due to its pro-oxidative, pro-inflammatory, and pro-fibrotic effects. MR is overexpressed in the adipose tissue of obese subjects, both in human and animal models, and several studies have suggested that this receptor may be involved in mediating obesity-related low grade chronic inflammation and metabolic abnormalities. The exact mechanisms underlying MR overexpression in obesity have not been fully clarified, but increased levels of ROS, induction of 11β-HSD1, and high levels of aldosterone have been proposed.

MRAs have been demonstrated to blunt the negative changes induced by adipocyte-MR activation, both in vitro and in animal models, displaying an anti-inflammatory and insulin-sensitizing effect. However, data regarding the specific effect of MR-blockade on obesity-related complications in humans are lacking. Therefore, further studies on accurately selected populations are needed to clarify the potential role of MRAs in this setting. 

## Data Availability

Not applicable.

## Abbreviations

11BHSD1: 11-β-hydroxysteroid-dehydrogenase type 1; 11BHSD2, 11-β-hydroxysteroid-dehydrogenase type 2; ATMs, adipose tissue macrophages; ATP, adenosine triphosphate; BAT, brown adipose tissue; BMI, body mass index; CLS, crown-like structure; FFA, free fatty acids; GR, glucocorticoid receptor; HFD, high fat diet; HIF1-α, hypoxia inducible factor 1-α; HOMA-IR, homeostasis model assessment for insulin resistance; IkB⍺, NF-kB inhibitor ⍺; IL-1β, interleukin-1β; IL-6, interleukin-6; IL-10, interleukin-10; MCP-1, monocyte chemoattractant protein-1; MR, mineralocorticoid receptor; MRAs, mineralocorticoid receptor antagonists; Myf-5, myogenic factor 5; NADPH, nicotinamide adenine dinucleotide phosphate; NF-kB, nuclear factor kappa-light-chain-enhancer of activated B cells; NLPR3, NLR family pyrin domain containing 3; NOX, NADPH oxidase; PAI-1, plasminogen activator inhibitor-1; PPAR-γ, peroxisome proliferator-activated receptor γ; ROS, reactive oxygen species; SAT, subcutaneous adipose tissue; siRNA, small interfering ribonucleic acid; TBARS, thiobarbituric acid reactive substance; TASK-1, Twik-related acid-sensitive K+ channel 1; T2DM, type 2 diabetes mellitus; TNF-α, tumor necrosis factor-α; UCP-1, uncoupling protein 1; VAT, visceral adipose tissue; VEGF, vascular endothelial growth factor; WAT, white adipose tissue.
